# Active case finding using mobile vans with artificial intelligence aided radiology tests and sputum collection for rapid diagnostic tests to reduce tuberculosis prevalence among high-risk population in rural China: Protocol for a pragmatic trial

**DOI:** 10.1371/journal.pone.0316073

**Published:** 2025-04-11

**Authors:** Xiaolin Wei, Dabin Liang, Zhitong Zhang, Kevin E. Thorpe, Lingyun Zhou, Jinming Zhao, Huifang Qin, Xiaoyan Liang, Zhezhe Cui, Yan Huang, Liwen Huang, Mei Lin

**Affiliations:** 1 Dalla Lana School of Public Health, University of Toronto, Toronto, Ontario, Canada; 2 Guangxi Zhuang Autonomous Region Center for Disease Control and Prevention, Guangxi Key Laboratory of Major Infectious Disease Prevention and Control and Biosafety Emergency Response, Nanning, Guangxi Zhuang Autonomous Region, China; The University of Georgia, UNITED STATES OF AMERICA

## Abstract

**Background:**

Tuberculosis (TB) remains a significant public health challenge, particularly in rural areas of high-burden countries like China. Active case finding (ACF) and timely treatment have been proven effective in reducing TB prevalence, but the impact on the TB epidemic when employing new technologies in ACF is still unknown. This study aims to evaluate the effectiveness of a comprehensive ACF package utilizing mobile vans equipped with artificial intelligence (AI)-aided radiology and GeneXpert testing in reducing TB prevalence among high-risk populations in rural Guangxi, China.

**Methods:**

A pragmatic cluster randomized controlled trial will be conducted in two counties of Guangxi, China. The trial will randomize 23 townships to intervention or control groups at approximately 1:1 ratio. The intervention group will receive an ACF campaign in Year 1 among high-risk populations, incorporating visited by mobile vans equipped with AI-based digital X-ray screening, symptom assessment, and sputum collection for GeneXpert testing. Control group participants receive usual care. TB patients identified in Year 1 will complete their treatment in Year 2. The primary outcome is the prevalence rate of bacteriologically confirmed TB among high-risk populations in Year 3. Process evaluation will explore acceptability, feasibility and adaptation of the intervention. We will conduct incremental costing study to inform future scale-up of the intervention in other settings.

**Discussion:**

This study will provide valuable insights into the effectiveness and feasibility of utilizing AI-equipped mobile vans and GeneXpert for TB ACF to reduce TB prevalence in rural settings. If successful, this model will contribute to possible solutions to achieve the WHO End TB Strategy by 2035.

**Trial registration:**

ClinicalTrials.gov NCT06702774

## Introduction

The United Nations passed the Resolution to end tuberculosis (TB) by 2035. TB is the leading cause of death in infectious diseases. In 2022, the world reported 7.5 million new TB cases and 1.3 million deaths caused by TB [1]. TB incidence has declined by 2% a year between 2010 and 2020. However, the rate has reverted to increase by 3.9% between 2020 and 2022 due to the COVID-19 pandemic [1]. To reach the END-TB resolution, global TB incidence has to be reduced to less than 100 per million by 2035 [2]. The world is looking for evidence of innovative approaches to eliminate TB in a relatively short time frame [3].

The principle of “prevent, search, detect, treat” has successfully reduced TB prevalence in high-income countries in the 20^th^ century [4]. Studies in high TB burden countries also proved that active case finding (ACF) at the community level would identify patients who are in their early stage of disease development, or who may be delayed or missed in the routine passive case finding at health facilities [4,5]. A 2009 study in rural Zimbabwe showed both mobile van and household-to-household sputum collection methods lowered TB prevalence by 41% in three years, showing the potential of using mobile vans in case finding [6]. Another study in rural Vietnam demonstrated that conducting annual community-based screening using a rapid molecular diagnostic tool (GeneXpert) for three years could reduce 40% of TB prevalence in a high TB burden setting [7]. However, GeneXpert costs around US$15 per test, which is prohibitive to be used as a large-scale screening tool.

More cost-effective active case-finding tools, such as computer-aided diagnostic tools using artificial intelligence (AI) deep learning neural networks, have shown satisfying sensitivity (86%) as a screening tool to reduce 66% of cases needed for laboratory tests [8]. Operational studies revealed the feasibility and effectiveness of using AI-facilitated X-ray and GeneXpert for community screening and diagnosis [9]; however, most studies were pre-after comparisons that were not properly designed to evaluate epidemiological impacts [10]. In addition, two studies in Africa highlighted that the interventions were more cost-effective when screening elders, close contacts of active TB patients, and individuals with comorbidities such as diabetes and HIV [11,12].

In this study, we aim to implement and evaluate the feasibility and effectiveness of a comprehensive intervention package in rural Guangxi, China, a high TB prevalent setting, that employs the latest technologies for active case finding to reduce TB epidemics among high-risk populations. The new technologies include employing a mobile van equipped with AI-aided radiology screening and GeneXpert test for active case finding in communities. Our study is designed as a pragmatic clustered randomized controlled trial to evaluate the effectiveness of the comprehensive package in reducing TB prevalence over a period of three years among high-risk populations.

## Methods

This protocol is reported according to the Standard Protocol Items: Recommendations for Interventional Trials (SPIRIT) guidelines (see S1 File).

### Study status and timeline

**Fig 1:**
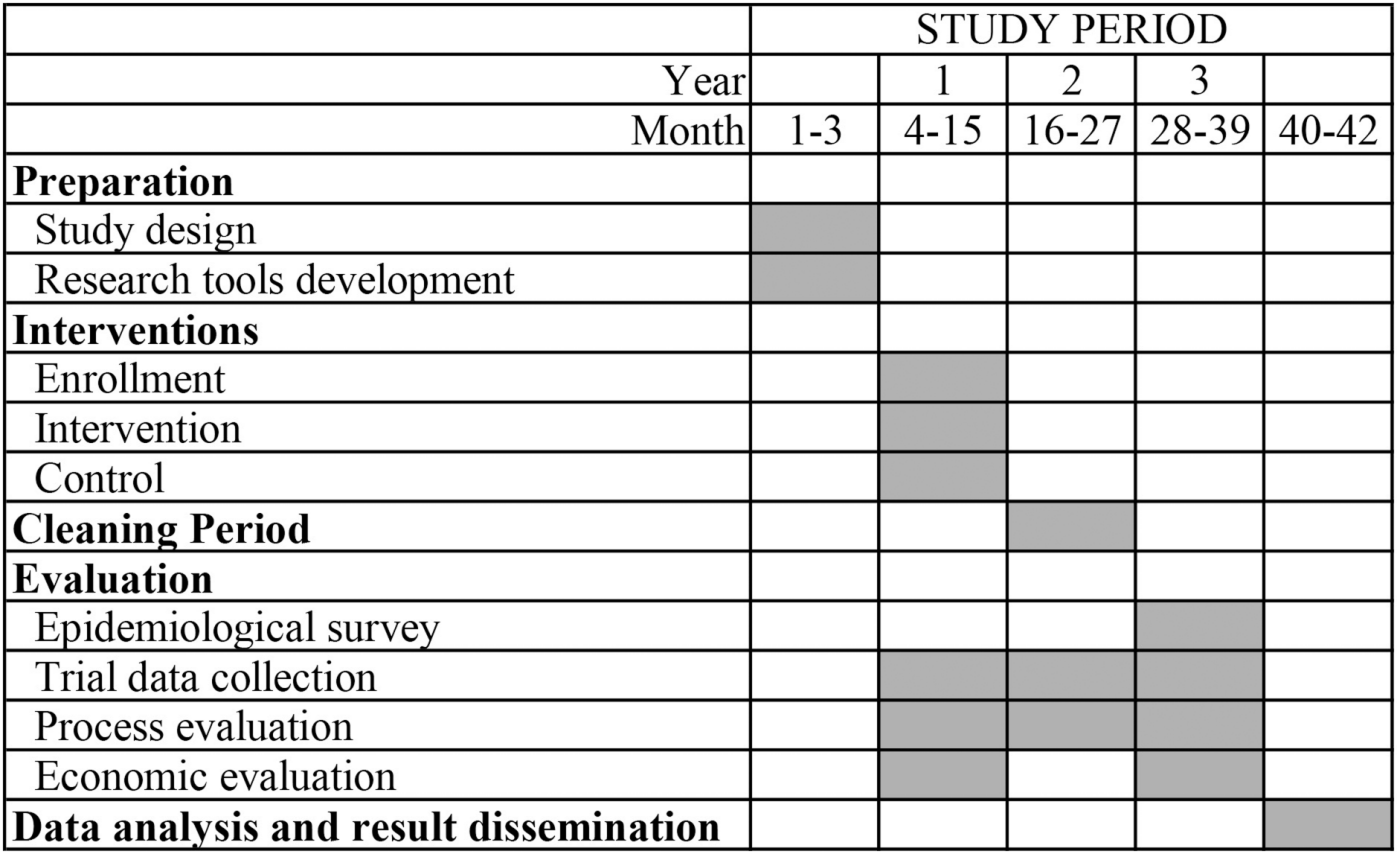
SPIRIT schedule of enrolment.

The lifespan of this study will be 42 months in total, including 36 months for the main trial (Year 1–3). More details of the trial timeline are included in Fig 1. The trial started in November 2021 and was suspended for 3 months due to COVID-19 control in Year 1. Thus, the progress was postponed accordingly. Participant recruitment and data collection have been ongoing by the time of submission of this protocol, which are estimated to be completed by the end of January 2025. The trial results will be disseminated through research articles and policy briefs after the trial evaluation is completed after April 2025. We aim to publish our results and findings in leading international medical journals and present at national and international conferences.

### Study design and setting

**Fig 2:**
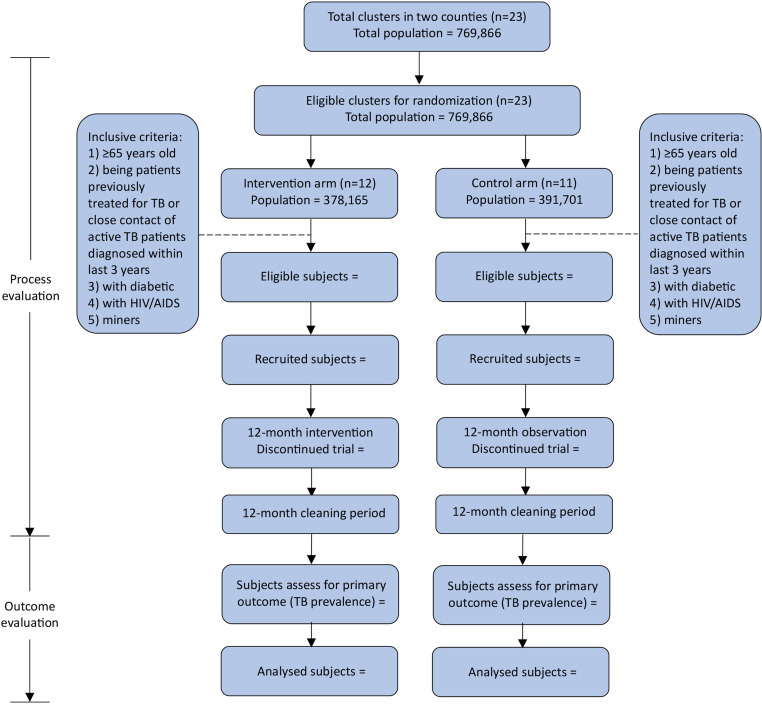
Trial design flow chart.

We will conduct a parallel two-group, cluster randomized controlled trial of a comprehensive package of active case finding followed by timely treatment in rural Guangxi China to evaluate whether the interventions could reduce the TB epidemic compared to the usual care (Fig 2). Guangxi is located in southwest China, bordering with Vietnam. Guangxi reported 31,913 TB cases with a notification rate of 70/100,000 in 2020 among all populations, representing the second highest TB burden in China. Of the reported TB cases, 74% were from rural areas, and 30% were elderly ( ≥ 65 years old). Mountainous areas in Guangxi had high TB notification rates; for example, the two trial counties (Xincheng and Xiangzhou of Laibin Prefecture) reported an average notification rate of 134/100,000, which is two times the provincial average. In addition, the Covid-19 pandemic has substantially delayed TB diagnosis and treatment, which would fuel up TB transmission and exacerbate the TB epidemic.

TB services in China are publicly funded and delivered by different public facilities at the county, prefecture, province and national levels. The TB free treatment policy covers basic TB diagnosis tests and treatment for drug susceptible cases, while health insurance schemes cover treatment costs for drug resistant cases. The County Centre for Disease Control (CDC) is responsible for TB planning, reporting and quality control, while the County Hospital provides clinical care. Passive case finding is the practice of usual care, where patients with chronic cough or other TB-related symptoms are self-presented or referred to the County Hospital for diagnosis and treatment. TB diagnosis is based on sputum smear, chest X-ray examinations, and clinical assessments. Standard WHO DOTS treatment regimen is provided for drug-susceptible cases. TB patients visit the County Hospital monthly to renew their medications. Patients take their anti-TB medications daily with the support of village doctors, who are community health workers. In Guangxi, village doctors are semi-private. They receive 50% of income from the township hospitals for their public health work such as maternal and child health, immunization, pap smear screening, and disease management including TB, diabetes, and hypertension, while they earn the other 50% income from clinical consultations covered under the rural health insurance scheme. According to the National TB Guideline, village doctors are responsible for visiting TB patients weekly. The public health doctor in the township hospital is responsible for supervising village doctors and visiting patients monthly. However, the actual frequencies of both doctors are far less and often substituted via phone calls. In each village, there is a female social worker in the Village Committee, who plays a major role in public education, maternal and child care, and protecting women’s rights. These female social workers have a strong connection with villagers but have not been involved in TB care.

### Eligibility criteria

#### Clusters.

Township is treated as the cluster of intervention. A township is an administrative unit with a township hospital and consists of 7–12 villages. All townships in Xincheng and Xiangzhou county, which provide their willingness to participate, will be included. Villages within the participating township serve as sampling units in the epidemiological survey at Year 3 to determine the TB prevalence rate.

#### Participants.

In each township, all permanent residents based on household registration who are 15 or older and reside in the village or township at the time of investigation become eligible if meeting one of the following criteria as high-risk population: 1) elderly (i.e., aged 65 and above), or 2) aged below 65 but with one of the following conditions: being patients previously treated for TB, or close contacts of a patient with a TB patient diagnosed within the last three years; having been clinically diagnosed with diabetes, HIV positive, or worked as a miner. All eligible participants must sign informed consent forms to be recruited. Residents who refuse participation will be excluded.

### Procedures

#### TB care provided in both groups.

Routine TB care is provided per the China National TB Guideline in both groups, where passive case finding is practiced. Patients self-present or are referred to the TB clinic at the County Hospital when they seek care for TB-related symptoms. TB diagnosis is based on X-ray examination, sputum smears and clinical assessment. Confirmed TB patients are treated in the TB Clinic of County Hospitals based on the WHO DOTS regimens for drug-sensitive TB. Those who are diagnosed with multi-drug resistant (MDR) TB are referred to prefecture level TB designated hospitals.

#### Usual care.

Usual care, i.e., the routine TB care provided in the National TB program, is provided in the control group, and no active case finding activities will be provided.

#### Intervention.

**Fig 3:**
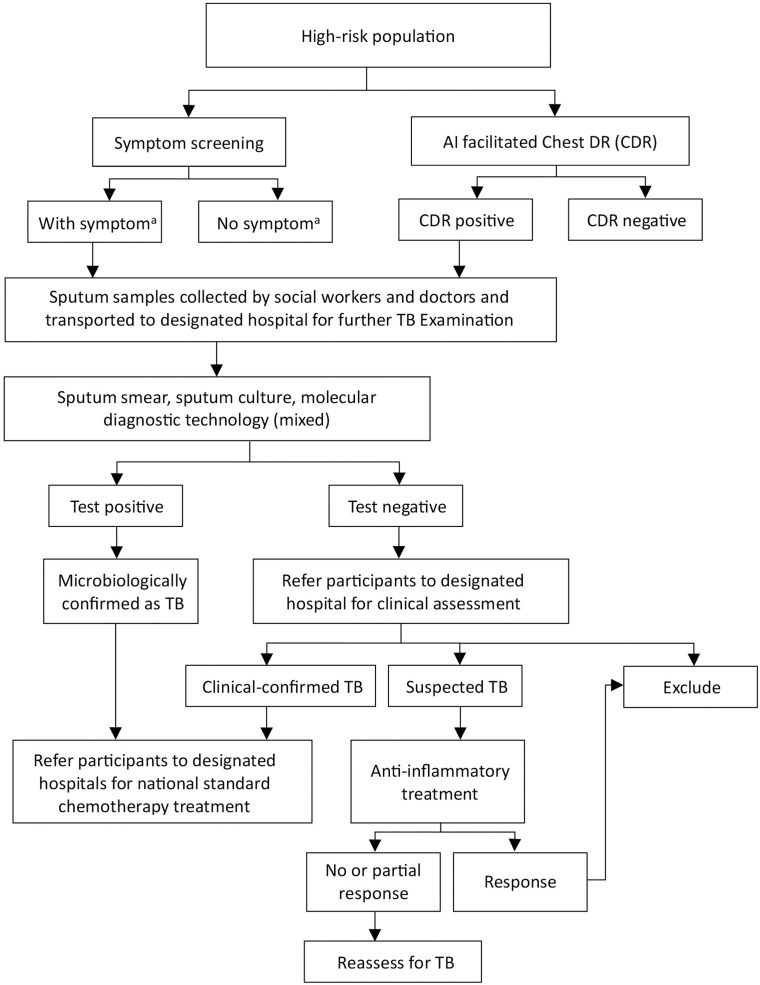
Algorithm of active TB case finding in intervention group. a. TB Symptoms include chronic cough over 2 weeks, hemoptysis, fever, night sweats, chest pain or unexplained weight loss. TB = tuberculosis, DR = digital radiography.

In the intervention group, in addition to usual care, we will implement an ACF campaign among all eligible participants in Year 1 (Fig 3). Prior to the visit, village social workers and village doctors will conduct door-to-door visits to recruit participants and obtain their informed consent. We will drive the mobile van to the village, and equip an AI-facilitated digital radiography (DR) machine and refrigerator on the van on a mutually agreed date. On the day, all eligible participants will be invited to complete a brief questionnaire regarding basic demographic information, medical history, smoking status and TB related symptoms, and then are invited to have their DR examined in the van. Based on digital chest X-rays, the AI software will identify TB assumptive participants who may have active TB, suspected TB or healed TB. In the first month of implementation in each county, one township hospital radiologist will facilitate the X-ray examination, double-check all results of the AI software, and make the final recommendation for presumptive TB. This process will be recorded to adjust the threshold score used by AI in each county. After the first month, the township hospital radiologist will still accompany the mobile clinic to help evaluate X-ray results if AI suggests for other lung diseases such as pneumonia or lung cancer, to minimize the risk of missing possible TB cases. Those who are presumptive of TB based on either DR examination or TB-related symptoms, i.e., any chronic cough over two weeks, hemoptysis, fever, night sweats, chest pain or unexplained weight loss, will be asked to produce a single on-site sputum. These villagers are also asked to take two specimen boxes for the collection of night and morning sputum. All staff are trained for good quality sputum sample collection, storage and transportation. Medical nebulizer inhalation will be provided to those who can not provide good-quality sputum. Sputum samples, either collected on the day or delivered by village doctors within 48 hours, will be stored in the refrigerator on the mobile van and transported back to the County Hospital daily for sputum testings, including smear microscopy, culture and GeneXpert assay. We will employ a sample mixing approach which has been validated in our previous publication [13]. Bacteriologically confirmed TB is defined by a positive result from any of the above sputum tests based on WHO and China National TB Program guidelines. Participants who are bacteriologically negative but have abnormal X-ray result or TB symptoms are referred to the county hospital for clinical assessment by a panel committee consisting of three senior doctors based on China National TB guideline for the diagnosis of clinically confirmed TB. All diagnosed TB patients, either bacteriologically or clinically confirmed, will be notified by the village doctor within 48 hours and referred to the County Hospital for standard WHO DOTS regimens per national guidelines.

#### Research activities apply to both groups:.

We will wait for a year (cleaning period) to ensure all TB patients identified in Year 1 complete the treatment. Due to the constraint of resources, we can not conduct ACF activities covering all villages in Year 3. Instead, we will conduct an epidemiological prevalence survey in the intervention and control groups at Year 3 using cluster-random sampling methods at the village level, adopted from WHO [14,15], targeting high-risk populations in both intervention and control groups, to compare the TB prevalence rates. Regarding the survey, we will apply the same active case-finding activities as outlined above in both intervention and control groups.

### Outcomes

Our primary outcome indicator is the prevalence rate of bacteriologically confirmed TB in Year 3 among the high-risk populations, including those 65 and older, those who are under 65 but have a history of tuberculosis treatment or have been in close contact with a person diagnosed with TB within the past three years, have been clinically diagnosed with diabetes, HIV, or have a background of working as a miner. According to WHO definitions, bacteriologically confirmed case is defined as having a positive test from any of the three tests (i.e., GeneXpert, smear or culture). Prevalence is estimated using a cluster-random survey among the high-risk populations to be conducted in both the intervention and control groups in Year 3. The numerator is the bacteriologically confirmed TB cases identified from the Year 3 prevalence survey plus TB cases reported from the National TB program, and the denominator is the number of high-risk populations who are permanent residents and reside in the area at the time of the prevalence survey. The numbers of numerators and denominators at the village level will be aggregated at the township level and calculated based on the WHO TB prevalence survey analysis guide to estimate the TB prevalence rate in the intervention or control group [14].

There is a list of secondary outcome indicators, which allow us to assess and evaluate whether our interventions could bring positive impacts on TB epidemic between the intervention and control groups: 1) prevalence rate of active TB of the high-risk populations, including both bacteriologically confirmed and bacteriologically negative but clinically confirmed cases, among the high-risk populations in Year 3. This indicator will be calculated based on findings from the prevalence survey in Year 3 estimated using the WHO TB prevalence survey guideline [14]; 2) notification rates of bacteriologically confirmed TB cases among the whole population in Year 3; and 3) notification rates of active TB cases, including both bacteriologically confirmed and clinically confirmed cases among the whole population in Year 3. Numerators of the two notification rates will include TB cases identified from our prevalence survey in Year 3 plus TB cases reported from the National TB Program, while both denominators include all adult permanent residents (i.e., those of 15 years and older) who reside in the intervention and control groups at the time of prevalence survey.

We will also collect costs from patient, health system and societal perspectives to conduct an incremental costing study. We will collect process indicators that include a) the proportion of bacteriologically confirmed cases identified by active case finding in the intervention group; b) the yield rate of our intervention, which is the number of bacteriologically confirmed cases per 100 persons screened using each strategy.

### Sample size

The sample size is calculated for the investigation of the primary outcome, i.e., the prevalence rate of bacteriologically confirmed TB, that will be collected in Year 3. We estimated that the TB prevalence rate among the high-risk populations in the trial area is about 300 per 100,000 based on the TB prevalence survey conducted in 2010 in Guangxi [16]. We assume our intervention will reduce the rate among the high-risk populations in the intervention group by 50% (i.e., to 150 per 100,000) within 24 months, which can be considered as a significant reduction at the population level from public health perspective. We estimate that the intra-class correlation coefficient for TB prevalence was 0.001 [7]. The average number of eligible participants is about 500 per village based on household registrations. We estimate that we would need 47 villages (i.e., approximately 23,500 participants) in each group to have 80% power to detect the reduction estimated above, assuming p <  0.05.

### Recruitment, randomization and blinding

There are 23 townships and 255 villages in Xincheng and Xiangzhou county of Laibin city [17]. We will implement the ACF activities at the township level to avoid contamination among villages because township hospitals manage village doctors. We seek to recruit all townships of the two counties. After recruitment, the study statistician will use a custom-written computer program to randomize all recruited townships, stratified by county, assigning them to the control and intervention group in an overall 1:1 ratio (12 intervention vs 11 control) [18]. Then township/village doctors and county hospital staff in the intervention group will be invited to receive training about specific interventions in this project. After randomization is done at the township level, the statistician will then randomly select 47 villages, stratified by county, from each group for the Year 3 prevalence survey. The selection of villages for prevalence survey will be sealed and blinded to providers and investigators until the Year 3 prevalence survey to prevent any provider-related bias. We will seek written informed consent from each participant before enrollment, as we need to collect their health information, bio-sample (sputum) and test result for research purpose.

Due to the nature of the intervention, neither single nor double blindness procedure of the allocation of groups is applicable. However, we will blind the data analysts who process the end-point data for outcome assessment.

### Data collection

We will collect patients’ basic information, disease history, health information, digital X-ray result, TB laboratory test results for TB, and clinical diagnosis, as well as routine TB reporting data. All data will be entered into a data-reporting website designed for this project, accessible only by designated researchers via local intranet with a password. Individual data will be de-identified and stored for five years after the completion of the trial.

#### ACF activities.

During the ACF, we will collect information about TB symptoms, including chronic cough for more than 2 weeks, hemoptysis, blood sputum, fever, night sweats, chest pain or unexplained weight loss, and TB risk factors (close contact history of tuberculosis patients, diabetes, AIDS/HIV and dust exposure). We will also collect the number of permanent residents of the village at the time of ACF, and the number of high-risk populations.

#### Laboratory testing and clinical consultation.

The county hospitals will carry out laboratory testing according to the ‘TB Laboratory Testing Protocol’. Sputum smear test, GeneXpert test and sputum culture are carried out at the same time. Every five samples of sputum will be mixed for GeneXpert test to save costs. Mixed samples that are positive will be re-tested individually to confirm the positive case. Country hospitals will distribute the laboratory testing results to patients within 48 hours. All TB diagnoses will be recorded in the ‘Register of Newly Diagnosed Patients’ in the National TB Program Registry. Clinical diagnosis based on the clinical assessment will be performed by a group of senior doctors in county hospitals according to the National TB Classification Criteria (WS196–2017) and Diagnostic Criteria (WS288–2017) [19,20]. We will collect TB cases diagnosed and reported in the National TB Program in both intervention and control groups through the three years.

#### Determination of the primary and secondary outcomes.

We will combine the TB cases identified from the Year 3 prevalence survey and TB cases reported from the National TB Program to determine the numerators of our outcomes. The data collection approach in Year 3 for the outcome evaluation will be the same as the ACF activities conducted in the intervention group in Year 1. We will use the TB diagnosis algorithm as defined in our ACF to determine the bacteriologically confirmed and clinically confirmed cases. We will also collect the routinely reported TB cases through the National TB Program for Year 3. We will collect the information of all registered permanent residents who reside in the village at the time of Year 3 survey to determine the relevant denominators.

### Statistical analysis

The results of this project will be reported according to the ‘Consolidated Standards of Reporting Trials: Extension for Cluster Trials’ (CONSORT) guidelines [21]. We will report a detailed statistical analysis plan later. In the full research paper, we will present appropriate descriptive statistics for the two groups and all relevant baseline cluster and participant characteristics. We will aggregate information collected in Year 3 prevalence survey at the township level to calculate primary and secondary outcomes. In addition, using cluster-level methods of analysis, we can get a main set of estimates for our primary and secondary outcomes after the trial, acting as the primary evidence for determining the effectiveness of this trial. Appropriate summary statistics and their associated 95% confidence interval for all differences outcomes between the intervention group and the control group will be calculated. No interim analyses are involved in this project.

For the primary outcome and all secondary rate-based outcomes (aggregated to the township level), the statistical analysis will employ a beta-binomial regression model. The intervention effect will be expressed as an odds ratio with 95% confidence intervals. Secondary analyses will attempt to analyze the patient-level data adjusted for age, sex, and ethnicity using a mixed-effects logistic regression model including random effects for township and village (to reflect the sampling strategy). In the event of non-convergence of the two-level model, models where township is included as a fixed effect rather than random will be employed.

For continuous secondary outcomes we will employ a linear mixed-effect model with random effects for township and village. This model will also adjust for the following variables: age, sex, and ethnicity. In the event of non-convergence of the two-level model, models where township is included as a fixed effect rather than random will be employed. The intervention effect will be expressed as the adjusted mean difference with 95% confidence intervals.

We will conduct subgroup analyses by county to reflect if there is any different effect due to environmental factors in each county. If the secondary patient-level analyses can be successfully employed, a sub-group analysis by sex will be attempted.

### Process evaluation

Since our project contains multiple components, the process evaluation during and after the trial is guided by the framework for the design and evaluation of complex interventions [22,23]. Utilizing a combination of qualitative and quantitative methods, process evaluation for the complex interventions in our cRCT can explore the mechanism of achieving the intervention effects [24]. In other words, we can get insights into a series of questions such as ‘What interventions are effective in which policies and implementation environments, for whom and why?’ We will conduct around 30 in-depth semi-structured interviews with leading personnel at all levels of health agencies, township and village doctors, county hospital staffs, social workers, and trial participants in the intervention group.

Our specific objectives for the process evaluation include: 1) to determine intervention accessibility by the number of subjects receiving screening, presented to county hospitals, and under treatment, as well as their feedbacks; 2) to explore the intervention effectiveness (reasons for both effectiveness and ineffectiveness), along with the relationship between interventions and expected results; 3) to access the acceptance of intervention implementation at the organization level and the staff level – any conflicts with existing management or policies, and corresponding adjustment; 4) to report intervention fidelity, the degree and quality of intervention completion, as well as challenges and unexpected results, at both subcluster-level and individual level.

#### Analysis of process evaluation.

All the recorded interviews will be transcribed and translated into English before qualitative analysis. We will use NVivo 10 software to process and analyze our qualitative data guided by a framework analysis as described by Gale at al [25], to identify themes related to our process evaluation objectives. The findings will be reported based on the Consolidated Criteria for Reporting Qualitative Research (COREQ) guidelines [26].

### Costing study

This study will measure the incremental cost-effectiveness of interventions compared with conventional TB finding, using research data and literature to evaluate both health system and social benefits. We will compare costs and outcomes of the intervention group to those of the control group throughout the 24-month trial. The objective of the costing study is to calculate the incremental cost-effectiveness ratio (the increased costs required for each 1% reduction in the prevalence of active TB among high-risk populations) using data from the project’s primary outcome.

#### Data collection.

We will send out a questionnaire to directors of all recruited township or village hospitals to collect health care resources used in the trial, along with their costs. The data include: 1) average salaries, in Renminbi (RMB), for each level of staff in designed hospitals who participated in this project and their estimate of time working on the project; 2) average salaries, in RMB, for township and village doctors who do not work in designated hospitals but still participated in the project and their estimate of time working on the project; 3) average salaries, in RMB, for social workers and other personnel worked in the project implementation and their estimate of time working on the project; 4) cost for TB screening (i.e., symptom screening, chest DR, mobile van operation, sending samples to county hospitals) and laboratory test (e.g., sputum smear, culture, and GeneXpert); and 5) indirect costs of ACF participants (loss of earnings due to participating in the screening, in RMB). To optimize the data quality, we will double-entry and random-check the input data.

#### Estimation of resources use and costs.

Healthcare resources in this case include resources used for TB detection and diagnosis. The cost per patient is calculated as the sum of consultation, screening and laboratory testing. The total costs is calculated for the main analysis population as described in the Statistical Analysis section [27].

#### Estimation of implementation costs.

The implementation costs are calculated separately and will not be accounted in the cost-effectiveness analysis. The objective of this estimation is to inform policy makers and stakeholder of the potential cost to facilitate their decision on whether to implement the intervention at scale. For each cluster, the implementation costs included the cost of training for personnel, the cost of personnel to attend training, the costs for developing and printing the handbook or guidelines per training attendee, operation cost of mobile van, driver cost, setup cost of AI facilitated DR, the costs for producing symptom questionnaires used in the project, and the costs of sending samples back to county hospitals.

### Trial management

Guangxi CDC will lead this project and have full access to all the anonymized data and information associated with the trial. We will establish a data monitoring committee (DMC) to ensure all data are collected in conformity with the agreed ethical guidelines, stored in Guangxi CDC properly and used for research purposes only. Meanwhile, the DMC is also responsible for the safety and privacy protection of all the subjects and providers participated in the trial. Additionally, there will be a trial steering committee (TSC) lead by external members to generate the progress of the trial. We will hold regular online meetings with both DMC and TSC every 6 months to get updates and discuss any protocol modifications, starting from the beginning of the study, until it completes. If there are any changes or concerns needed to report or discuss, the committees can meet on an ad hoc basis with the co-leaders as well. Moreover, a trial management unit (TMU) will be established in consisting of three local staff. The responsibility of the TMU is to better manage the day-to-day activities of the trial according to the study protocol.

### Ethical approvals and consent to participate

The study has obtained ethical approval from the Guangxi Institutional Review Board of the Guangxi CDC on 26 August 2021 (Ref: GXIRB2021–0033, see S2 File for the original approved proposal). Written informed consent form will be collected before any patient is recruited into the study.

## Discussion

This study aims to enhance the existing TB control protocol in an area of high TB prevalence. As the location of implementation, Laibin is an ethnically diverse area with the highest reported TB incidence rate in Guangxi region. In other words, it has an urgent need to find a more effective way to manage its TB epidemic. Given that about 27% of all reported cases in Guangxi region are from the elderly population, the interventions target the elderly and also pay special attention to other populations who have high risk of TB infection [17,28,29].

The study has significant policy relevance to China and globally. The objective of this study is to effectively reduce the TB epidemic in Guangxi and achieve the WHO’s End TB Strategy [2] milestones by 2024. If successful, the prevalence of TB among high-risk populations in the intervention areas will be reduced by more than 50%. It aligns with the goal to build a healthy China as well. The successful implementation of this study will yield a feasible TB control strategy that can be replicated in other parts of China, which may provide a model for countries with similar contexts to achieve the WHO’s goal to end TB by 2035 [2].

Furthermore, active screening interventions involve innovative technologies. Since over 40% of areas in Laibin are mountainous, the mobile health vehicles equipped with DR machines and refrigerators make active screening procedures more convenient, and all the bio-samples can be properly stored and timely transported to the county hospital. In addition, the AI-based diagnostic system has been verified (above 90% sensitivity and 70% specificity) which offers axillary support to detect abnormalities in DR images, increasing the test efficacy [30].

Nevertheless, there are some limitations in this study as well. First, TB-suspected participants in the active screening intervention are required to collect two sputum samples (one for morning and one for night sputum) and send them to village doctors. Although specific equipment and instructions will be given, there are still risks of improper sample collection or transportation, which may affect laboratory results and TB diagnosis. We provide training for people assumptive of TB in sputum collection to improve the quality. Second, due to the limitation of funding and human resources, we are not able to conduct active case finding among all populations in participating townships in Year 3. Therefore, the trial cannot evaluate the impact of interventions on TB prevalence in the general population. On the other side, a prevalence survey including the whole population may not be feasible anyway because most people of working ages in rural areas moved to urban areas as migrants during most time of the year, while only elders, who comprise the majority of our high-risk population, and children stay at home. Our active case-finding activity targeting high-risk populations would significantly reduce TB in rural areas. Third, there is a possibility of participants migrating between villages. However, this situation is rare due to local culture and similar income level among villages. This was confirmed by another trial we conducted in Guangxi [31]. Lastly, we are not able to conduct a TB prevalence survey before the start of interventions to serve as a baseline due to limited resources. We believe the randomization procedure will minimize any confounding effects from the baseline effect in the two groups.

## Supporting information

S1 File SPIRIT checklist. (PDF)

S2 File Protocol approved by ethics committee. (PDF)

## Abbreviations

AI: artificial intelligence
AIDS: acquired immunodeficiency syndrome
CDC: Center for Disease Prevention and Control
DMC: Data Monitoring Committee
DR: digital radiography
HIV: human immunodeficiency virus
MDR-TB: multidrug-resistant tuberculosis
NTP: National TB Program
RCT/cRCT: randomised controlled trial/cluster randomised controlled trial
RMB: renminbi
TB: tuberculosis
TCS: Trial Steering Committee
TMU: trial management unit
WHO: World Health Organization.
