# Supplementary material for: Active case finding using mobile vans with artificial intelligence aided radiology tests and sputum collection for rapid diagnostic tests to reduce tuberculosis prevalence among high-risk population in rural China: Protocol for a pragmatic trial
Source: PLoS One. 2025 Apr 11;20(4):e0316073. doi: 10.1371/journal.pone.0316073 (PMC11990735; doi:10.1371/journal.pone.0316073)
Supplement: S2 File — (PDF) [file pone.0316073.s002.pdf]

广西结核病高发地区遏制流行干预关键技术研究实施方案 (v20210823)

一、项目技术需求背景、立项依据与必要性

结核病是严重危害广大人民群众身体健康和生命安全的重大传染性疾病，是因病致贫、因病返贫的重要原因之一。近年来，广西结核病防治服务能力不断提高，疫情呈现明显下降的趋势。2018-2020 年全区的报告发病率和发病数均呈下降趋势，但由于感染结核菌的人群基数大、结核病发现难、疗程长等多种因素，2020 年全区的结核病疫情在全国排第七位，防控形势依然严峻。肺结核报告发病率最高为来宾市，2018-2020 年的报告发病率分别为 145.05/10 万、152.16/10 万和 133.71/10 万，远远高于全区的平均水平（82.82/10 万、81.12/10 万和 70.39/10 万）。

广西结核病患者 74%为农村患者，其中 30%为 65 岁以上老年人，其报告发病率（191.38/10 万）远高于全区报告发病率（70.39/10 万），老年人患者发病数占全区患者发病数的 27.14%（9476/34913）。由于部分肺结核患者症状不典型、广西山区较多就医不便以及群众就医意识不高等多种原因，患者不能得到及时的诊断和治疗。因此，目前老年人的活动性肺结核报告发病率可能被低估，还有较多传染源没有被发现和治疗，极不利于本地结核病的控制。

肺结核主动发现，指由卫生主管部门或医疗卫生保健单位为发现肺结核患者而组织社区等人群接受与肺结核有关的医学检查。老年人群进行肺结核主动筛查的方式主要包括，通过填写问卷或医生问诊进行症状筛查，胸部 X 线摄影检查，痰涂片痰培养检查及 GeneXpert MTB/RIF 分子诊断技术等。近年来，随着人工智能影响诊断技术的

发展，筛查手段有了更多选择，使用AI技术进行胸片筛查可以减少人为判断的错误，同时减少现场所需要的专业影像技术人员，适宜在专业技术人员缺少的基层使用，在人群筛查中提高检查效率及降低人力成本。

非洲的两项研究亦表明成本-效益较好的主动筛查策略包括对老年人群先进行问卷调查，对症状可疑者进行胸部X线摄影检查和痰涂片检查。主动筛查策略的成本一效益还受到筛查地区及筛查选取人群等因素的影响，当开展主动筛查的地区结核病患病率较高时，选取的筛查人群为 65 岁及以上的老年人、肺结核患者的密接者、糖尿病患者等，所产生的成本-效益更高。

综上所述，为了及早发现和治疗管理老年人等重点人群中的结核病人，进一步降低广西高疫情地区结核病负担，本研究拟在来宾市的忻城和象州 2 个高疫情县开展结核病主动筛查，积极发现和治疗管理肺结核患者以降低结核病疫情。

二、项目研究目标与内容

2.1 研究目标

在广西来宾市开展结核病主动筛查，积极发现和治疗管理肺结核患者，有效降低结核病疫情，实现世界卫生组织终止结核病策略阶段性目标，形成应用适宜新技术，在中国和世界可复制、可推广的终结结核综合干预模式，为全球终结结核病提供理论依据。

2.2 研究的主要内容

（一）设计符合广西卫生资源条件的综合干预服务包，包括：使用流动车进村，以人工智能结核影像辅助诊断系统对研究区域的 65 岁以下重点人群（近 3 年内确诊

的活动性肺结核患者及其密切接触者、糖尿病患者、艾滋病患者及矿工）和 65 岁及以上全部老年人口进行肺结核病主动筛查。

（二）以实施性研究的综合评价手段对干预效果及其对实际工作和卫生政策影响进行科学评价，包括：利用整群随机对照试验对主要效果指标进行评价；利用过程评价方法为项目方案的修订和优化提供依据，同时判断干预效果与不同干预手段和实施过程的关系，深入研究干预措施起作用的原理；开展成本效果分析对今后大规模干预带来的健康经济效益做出评价，为在广西自治区开展全区消灭结核病提供一套切实可行的方法。

2.3 研究方法

2.3.1 研究设计：整群随机对照试验

2.3.2 项目选址

选择在来宾市的忻城和象州两个县的所有乡镇开展。项目采用分层随机化方式平衡组间人口特征差异，综合考虑研究人口数量，在每县采用 1:1 随机化分组，最终选定 12 个干预乡镇，11 个对照乡镇。

2.3.3 研究对象：主动肺结核病筛查对象为 65 周岁以下重点人群（近 3 年内确诊的活动性肺结核患者及其密切接触者、糖尿病患者、艾滋患者及矿工）和 65 周岁及以上的全部人群。两组符合要求的研究对象预计共约 72000 人。

2.3.4 研究实施流程：

（1）项目实施方案制定及工作动员：（3 个月）

制订实施方案细则，准备研究材料和干预工具，落实检测工具、资金和人员的到位情况，组织项目启动会，发动各级卫生部门配合开展项目工作。

（2）开展干预工作：（12 个月）

在干预组乡镇开展以下工作，对照组乡镇同期按照现有结核病管理要求进行常规管理工作，不实施干预：

1） 社区主动肺结核病筛查：干预组乡镇卫生院针对全部研究对象采用流动健康车形式，提供 1 次肺结核病主动筛查服务，在筛查工作开始前需先获得研究对象签署的知情同意书。通过问诊，以问卷形式收集筛查对象的可疑症状以及结核病重点人群信息（结核病诊断史、结核患者密切接触史、糖尿病、AIDS/HIV 及粉尘暴露）。对有慢性咳嗽≥2 周，咯血、血痰，或发热、盗汗、胸痛或不明原因体重减轻的研究对象标记为肺结核可疑症状者。之后所有研究对象在流动健康车上进行胸片筛查，医务人员通过人工智能结核影像辅助诊断系统快速判断筛查对象胸片异常情况。对所有疑似肺结核患者（有可疑症状或胸片异常），由专业人员指导，在指定隔离的通风位置取痰，现场给需要的人提供雾化引痰设备，并给与夜间痰、晨痰痰盒共 2 个，并于第二天中午前由村医送至流动健康车。留痰点发放的痰盒应标明姓名、编号，告知对象留取合格痰标本的方法。流动健康车配备独立冰箱，完善生物安全方案，存放采集的痰液并于 48 小时内运输至定点医院，同时也将需要转诊的受检对象的胸片拷贝至定点医院。

对于胸片异常或者肺结核可疑症状者在鉴别诊断的基础上，填写“双向转诊单”。推荐其到定点医院进行结核病检查，责任落实到乡医和村医，确保其及时就医，并且在 1 周内进行电话随访。对于实验室确诊病人需要在 24 小时内通知，48 小时内对密接人员完成检查，并保证病人 48 小时内去定点医院就诊。

2） 定点医院结核病检查：开辟绿色通道，社区筛查转诊的患者直接就诊。

① 如有需要补充拍摄 DR 胸片及收集痰液：对于社区筛查拷贝过来的胸片有缺失或者质量不佳的需要补充拍摄 1 张胸部正位片诊断，有必要者可加摄 1 张侧位片诊断，胸片上标注受检者编号和姓名。胸片质量如果影响诊断或鉴别诊断时，应及时重拍。对于社区筛查收集痰液送至定点医院时发现缺失或者质量不合格的，需给患者开具痰检单，到医院留痰点再次留即时痰，并发夜间痰、晨痰痰盒共 2 个，让受检者第二天带回。

② 实验室补充检测：实验室根据《结核病实验室检验规程》规定开展工作，按照痰检单登记《痰涂片检查登记本》（标注受检者编号），对收集到的痰样本当天完成涂片检查，2 日内完成结果报告，同时开展痰培养和 GeneXpert 检测（建议对 5 份痰合并混检，有阳性者将 5 份再单独进行 GeneXpert 检测）。痰检结果出来后通过乡医和村医告知患者，并确保需要就诊的患者在 48 小时内到定点医院就诊。

③ 临床诊断：临床医生结合临床症状和检查结果进行临床诊断，诊断结果详细登记在《初诊患者登记本》（标注受检者编号）。肺结核诊断执行国家 2017 年版结核病分类标准（WS196-2017）、诊断标准（WS288-2017）。

a. 排除结核诊断：登记怀疑诊断如“肺炎”、“支气管炎”等，建议患者进一步治疗处理；对疑似矽肺等可能涉及职业病诊断的，不出具诊断意见，仅建议患者至专业机构定诊；临床诊断肺结核合并矽肺的，按肺结核诊断处理，矽肺建议患者至专业机构定诊。

b. 对于诊断为活动性肺结核（初、复治）的，通知乡镇卫生院、社区卫生服务中心追踪患者到定点医院按照国家标准化疗方案进行治疗，纳入规范治疗管理。对于涂阴临床诊断肺结核的，注意区分是否在治、是否新近完成治疗、不规律治疗情况，追

踪患者到定点医院完善检查，按照国家标准化疗方案纳入治疗管理。

c. 对于疑似肺结核诊断的，需要进行诊断性抗炎治疗者，给出抗炎治疗建议，可根据患者意愿在乡镇卫生院开展治疗观察，抗炎治疗后要求追踪患者到定点医院复查，需要抗结核诊断性治疗者，追踪患者到定点医院诊治。对诊断为非结核患者，告知患者病情，推荐患者至专业机构进一步诊断治疗。

（3）结核病患者治疗：（12 个月）对干预组通过主动筛查发现的患者追踪并完成治疗工作。

（4）终线数据收集：（12 个月）

1）流行病学调查：在干预组和对照组乡镇的全部研究对象通过随机抽样开展流行病学调查，对比干预结束后两组活动性菌阳肺结核病的流行率。调查方法同干预阶段的筛查和诊断流程。根据 2010 广西流调数据，预计对照组研究对象中活动性菌阳肺结核流行率为 300/10 万，项目预期将干预组研究对象的活动性菌阳肺结核流行率降低一半以上，即 150/10 万，采用α双侧 0.05，检验功效 80%，组内相关系数 0.001，计算所需调查样本为 47000 人，共需随机选取 94 个村（干预组 47 个，对照组 47 个），平均每个村需调查 500 例研究对象，选中的村人口不足的由北向临近村补充。

（5）研究评价（3 个月）

1）首要指标为第三年研究对象的实验室确诊肺结核流行率（菌阳）。次要指标包括：① 第三年研究对象的活动性肺结核流行率（包括菌阳及菌阴确诊结核病患者）；②第三年全人群实验室确诊肺结核的报告发病率（菌阳）；③ 第三年全人群的活动性肺结核的报告发病率（包括菌阳及菌阴确诊结核病患者）。对比例度量的指标统计分析将采用混合效应逻辑回归模型，对连续型数据指标采用线性混合效应模型，均考虑

乡镇和村庄的随机效应，并在分析中调整年龄、性别及民族变量。

2）过程评价：根据复杂干预设计和评价框架，采用定性定量结合的方法对整群随机对照试验的复杂干预进行评价，用以了解干预效果达成的机制，即哪些干预措施在什么政策和实施环境中起了效果，受众反馈如何，是如何起效的。过程评价通过现场观察记录、项目执行数据收集以及针对各级卫生机构领导人员、参与结核病筛查和治疗管理的医务人员、实验室人员以及参与筛查的当地居民进行访谈共计约 30 人进行深入定性访谈，来了解 1）干预措施的可及性：筛查和治疗的人数；2）效果：干预措施与预期结果之间的关系，探索起效或无效的原因；3）可接受性：干预措施执行时在机构层面和工作人员层面相应的调整和原因，干预措施如何同现有日常管理工作相结合，有无冲突的地方；4）实施：干预措施完成的程度和质量，面临的困难和挑战以及未预计到的结果。

3）卫生经济评价：本研究将同常规结核病的发现相比，测算干预措施的成本效益，通过研究数据和文献数据同时从卫生系统以及社会效益两个角度进行评估。研究将收集干预组与对照组相比增加的成本，结合项目首要指标数据计算增量成本—效果比（即活动性肺结核流行率每降低 1%所需要增加的成本）。

三、质量控制：

1. 培训：自治区疾控中心组织专家对现场参与成员进行统一培训，对筛查工作进行详细讲解和模拟练习，全面掌握此次结核病主动筛查的具体内容和方法。同时，自治区疾控中心指导定点医院根据项目工作开展的反馈与实际情况，不定期地组织开展现场培训，以保证现场实施工作的项目质量，及时解决问题。

2. 现场质量督导：（1）自治区和来宾市在项目启动及项目开展中和数据验收时对县、乡均督导一次，必要时加强督导；（2）来宾市在筛查期间对乡镇每月督导一次，项目开展期间可按需增加督导频次；（3）每次督导检查后，督导组要撰写督导报告，并向自治区项目组反馈。

3. 数据核查：在完成筛查当天，资料管理员检查现场完成的个人信息表中的筛查栏目有无缺项，是否有逻辑错误。如有问题第二天要及时与筛查员联系进行改正。

Translated Copy

Research Protocol for Key Technologies to Control Tuberculosis Epidemics in High-Prevalence Areas of Guangxi （v20210823）

1. Background, Justification, and Necessity of the Project

Tuberculosis (TB) is a major infectious disease that significantly endangers public health and life safety. It is also a key cause of poverty due to illness. In recent years, Guangxi’s capacity to prevent and control TB has improved, leading to a noticeable decline in reported incidence and case numbers from 2018 to 2020. However, challenges persist due to the large population at risk, difficulties in early detection, and prolonged treatment durations. In 2020, Guangxi ranked seventh nationally in TB incidence, with a particularly severe situation in Laibin City, where the reported TB incidence rates in 2018–2020 were 145.05/100,000, 152.16/100,000, and 133.71/100,000, respectively—substantially higher than the provincial averages (82.82/100,000, 81.12/100,000, and 70.39/100,000).

Rural residents comprise 74% of TB cases in Guangxi, with 30% of these being individuals aged 65 and older. The reported incidence among elderly individuals (191.38/100,000) far exceeds the provincial average (70.39/100,000). Elderly cases represent 27.14% of all reported cases (9,476 out of 34,913). Challenges such as atypical symptoms, limited access to healthcare in mountainous regions, and low health-seeking awareness contribute to underdiagnosis and delayed treatment. Consequently, the active pulmonary TB incidence

among the elderly is likely underestimated, leaving undetected transmission sources, which hinders TB control.

Active case-finding (ACF) for TB involves health authorities or healthcare units organizing community-based medical examinations to detect TB cases. For elderly populations, screening methods include symptom questionnaires, chest X-rays, sputum smear and culture tests, and molecular diagnostic techniques such as GeneXpert MTB/RIF. Advancements in artificial intelligence (AI) now allow AI-assisted chest X-ray screening, reducing human error and the need for specialized imaging personnel, making it suitable for use in resource-limited settings.

Studies in Africa have demonstrated that cost-effective ACF strategies include symptom screening followed by chest X-rays and sputum smear tests for individuals with suspected symptoms. The cost-effectiveness of ACF is influenced by factors such as TB prevalence in the region and the population targeted for screening. When conducted in high-prevalence areas and among high-risk groups (e.g., elderly individuals, close contacts of TB patients, diabetics), the cost-effectiveness improves significantly.

In summary, to detect and manage TB patients early in key populations like the elderly and further reduce the TB burden in high-prevalence areas of Guangxi, this study plans to conduct ACF in Xincheng and Xiangzhou counties of Laibin City, actively identifying and managing pulmonary TB patients to lower the TB epidemic.

2. Research Objectives and Content

2.1 Research Objectives

To conduct ACF in Laibin City, Guangxi, actively identify and manage pulmonary TB patients, effectively reduce the TB epidemic, achieve the phased goals of WHO's End TB Strategy, form an applicable comprehensive intervention model replicable and scalable within China and globally, providing theoretical basis for global efforts to end TB.

2.2 Main Research Contents

(a) Designing a comprehensive intervention service package suited to Guangxi's health resource conditions, including: using mobile health vehicles to enter villages and employ an AI-assisted TB imaging diagnostic system to screen for pulmonary TB among key populations under 65 years old (confirmed active pulmonary TB patients within the past three years and their close contacts, diabetic patients, AIDS patients, miners) and all elderly people aged 65 and above in the research area.

(b) Scientifically evaluating the impact of the intervention and its effects on actual work and health policies using implementation science evaluation methods, including: utilizing cluster randomized controlled trials to evaluate primary effectiveness indicators; process evaluation methods to provide evidence for revising and optimizing project plans while analyzing the relationship between intervention effects and different intervention methods and implementation processes, deeply studying the principles behind how interventions work; conducting cost-effectiveness analysis to evaluate the health economic benefits of large-scale future interventions, providing a practical method for eliminating TB throughout Guangxi Autonomous Region.

2.3 Research Methods

2.3.1 Study Design: Cluster Randomized Controlled Trial

2.3.2 Project Location Selection

The project will be conducted in all townships of Xincheng and Xiangzhou counties in Laibin City. A stratified randomization method will be used to balance demographic characteristics between groups, taking into account the study population size, and each county will be randomly assigned in a 1:1 ratio, ultimately selecting 12 intervention townships and 11 control townships.

2.3.3 Study Subjects: Active pulmonary TB screening and treatment subjects include key populations under 65 years old (confirmed active pulmonary TB patients within the past three years and their close contacts, diabetic patients, AIDS patients, miners) and all individuals aged 65 and above. It is estimated that approximately 72,000 eligible subjects will be included in both groups.

2.3.4 Implementation Process:

(1) Formulation of Implementation Plan and Mobilization Work: (3 months)

Formulate detailed implementation plan, prepare research materials and intervention tools, ensure the availability of testing equipment, funds, and personnel, organize a project launch meeting, and mobilize all levels of health departments to cooperate in project work.

(2) Conduct Intervention Work: (12 months)

In the intervention group townships, the following activities will be carried out. Control group townships will follow existing TB management requirements without implementing

interventions during the same period:

a) Community-based active pulmonary TB screening: Mobile health vehicles will provide one-time pulmonary TB screening services to all research subjects in intervention group townships. Before screening begins, informed consent must be obtained from research subjects. Questionnaires will be used to collect information on suspected symptoms and key population details (history of TB diagnosis, contact with TB patients, diabetes, AIDS/HIV, dust exposure). Individuals with chronic cough ≥2 weeks, hemoptysis, blood-streaked sputum, or fever, night sweats, chest pain, or unexplained weight loss will be marked as suspected pulmonary TB cases. All research subjects will undergo chest X-ray screening on mobile health vehicles, and medical staff will quickly judge chest X-ray abnormalities using an AI-assisted TB imaging diagnostic system. For all suspected pulmonary TB cases (with symptoms or abnormal chest X-rays), professionals will guide them to collect sputum samples in isolated, ventilated locations, provide nebulizers for those who need it, and issue two sputum containers for nighttime and morning samples, which village doctors will deliver to the mobile health vehicle by noon the next day. Sputum containers should be labeled with names and numbers, and instructions given on proper sample collection. Mobile health vehicles will be equipped with independent refrigerators, implement biosafety protocols, store collected sputum, and transport it to designated hospitals within 48 hours. Chest X-rays of referred individuals will also be copied to designated hospitals.

For individuals with abnormal chest X-rays or suspected pulmonary TB symptoms, after differential diagnosis, "two-way referral forms" will be filled out. They will be recommended

to visit designated hospitals for TB examination, with responsibilities assigned to township and village doctors to ensure timely medical visits, and follow-up calls made within one week. Confirmed laboratory-diagnosed patients should be notified within 24 hours, and close contacts checked within 48 hours, ensuring patients visit designated hospitals within 48 hours.

b) TB Examination at Designated Hospitals: Green channels will be established for community-screened referrals for direct consultation.

i. If additional DR chest X-rays or sputum collection are needed: If the chest X-ray copied from the community has missing parts or poor quality, an additional anteroposterior chest X-ray will be taken, and if necessary, a lateral view. If the image quality affects diagnosis or differential diagnosis, it should be retaken promptly. For sputum samples delivered to designated hospitals from the community found to be missing or of poor quality, patients will be asked to provide immediate sputum again at hospital collection points, along with two containers for nighttime and morning samples to take home.

ii. Laboratory supplementary testing: Laboratories will operate according to the "Laboratory Testing Procedures for Tuberculosis," registering sputum smear examination results in the "Sputum Smear Examination Register" (annotated with subject numbers). Collected sputum samples will be examined on the same day, with results reported within two days. Simultaneously, sputum cultures and GeneXpert tests will be conducted (suggest pooling five samples for initial testing, and if positive, individually retesting the five samples with GeneXpert). Results will be communicated to patients via township and village doctors, ensuring patients needing treatment visit designated hospitals within 48 hours.

iii. Clinical Diagnosis: Combining clinical symptoms and test results, clinical doctors will make clinical diagnoses, which will be meticulously recorded in the "First Visit Patient Register" (annotated with subject numbers). Pulmonary TB diagnosis will follow the national 2017 edition of TB classification standards (WS196-2017) and diagnostic criteria (WS288-2017).

A. Exclude TB diagnosis: Record suspected diagnoses such as "pneumonia" or "bronchitis" and recommend further treatment. For suspected silicosis or other potential occupational diseases, no diagnosis opinion will be issued, only recommending patients seek diagnosis at specialized institutions. For clinically diagnosed pulmonary TB combined with silicosis, handle as pulmonary TB, recommending patients seek silicosis diagnosis at specialized institutions.

B. For diagnosed active pulmonary TB (initial or retreatment), notify township health centers and community health service centers to track patients to designated hospitals for treatment according to standardized national chemotherapy regimens and include them in standardized treatment management. For smear-negative clinically diagnosed pulmonary TB, distinguish whether they are currently in treatment, recently completed treatment, or have irregular treatment histories, tracking patients to designated hospitals for completion of examinations and inclusion in treatment management according to standardized national chemotherapy regimens.

C. For suspected pulmonary TB requiring diagnostic anti-inflammatory treatment, provide anti-inflammatory treatment suggestions, potentially treating in township health centers with

patient consent, and require tracking patients for re-examination at designated hospitals. For those requiring anti-TB diagnostic treatment, track patients to designated hospitals for treatment. Inform non-TB patients of their condition and recommend further diagnosis and treatment at specialized institutions.

(3) TB Patient Treatment: (12 months) Track and complete treatment for patients identified through active screening in the intervention group.

(4) Endpoint Data Collection: (12 months)

a) Epidemiological Survey: Conduct epidemiological surveys on all research subjects in intervention and control group townships through random sampling, comparing the prevalence of active smear-positive pulmonary TB between the two groups after the intervention ends. The survey method will be similar to the screening and diagnostic procedures during the intervention phase. Based on 2010 Guangxi survey data, it is expected that the prevalence of active smear-positive pulmonary TB in the control group will be 300/100,000, with the project aiming to reduce this to less than half in the intervention group, i.e., 150/100,000. Using a two-sided α of 0.05, power of 80%, intra-cluster correlation coefficient of 0.001, the required survey sample size is calculated to be 47,000 people, totaling 94 villages (47 in the intervention group and 47 in the control group), averaging 500 subjects per village, with insufficiently populated villages supplemented by nearby villages to the north.

(5) Research Evaluation (3 months)

a) Primary Indicator: Laboratory-confirmed prevalence of pulmonary TB (smear-positive) in

the third year. Secondary indicators include: ① Prevalence of active pulmonary TB (including smear-positive and smear-negative confirmed TB patients) in the third year; ② Reported incidence of smear-positive laboratory-confirmed pulmonary TB in the third year for the entire population; ③ Reported incidence of active pulmonary TB (including smear-positive and smear-negative confirmed TB patients) in the third year for the entire population. Statistical analysis of proportion measures will use mixed-effects logistic regression models, and continuous data indicators will use linear mixed-effects models, considering random effects of towns and villages and adjusting for age, gender, and ethnicity variables in the analysis.

b) Process Evaluation: Based on the framework for designing and evaluating complex interventions, qualitative and quantitative methods will be combined to evaluate the complex intervention of cluster randomized controlled trials, understanding the mechanisms of achieving intervention effects, i.e., which intervention measures worked under what policy and implementation environments, audience feedback, and how they worked. Through on-site observation records, data collection from project implementation, and in-depth qualitative interviews with approximately 30 individuals, including leaders of health institutions at various levels, medical staff involved in TB screening and treatment management, laboratory personnel, and local residents participating in the screening, the evaluation will understand: 1) Accessibility of interventions: the number of screenings and treatments; 2) Effectiveness: the relationship between intervention measures and expected outcomes, exploring reasons for effectiveness or ineffectiveness; 3) Acceptability:

adjustments and reasons at the institutional and staff levels when implementing intervention measures, how they integrate with existing daily management work, and any conflicts; 4) Implementation: the degree and quality of completion of intervention measures, challenges faced, and unexpected results.

c) Health Economics Evaluation: Compared with conventional TB discovery and treatment management, this study will calculate the cost-effectiveness of intervention measures. By collecting data from the intervention and control groups and combining project primary indicator data, incremental cost-effectiveness ratios (the cost increase required to reduce the prevalence of active pulmonary TB by 1%) will be calculated.

3. Quality Control:

1. Training: Experts from the provincial CDC will train on-site participants uniformly, explaining and practicing screening work in detail to fully master the specific content and methods of this ACF. The provincial CDC will also organize on-site training as needed based on project feedback and actual circumstances to ensure project quality and address issues promptly.
2. On-site Quality Supervision: (1) Provincial and municipal supervision once at project start, during implementation, and upon data acceptance, with increased frequency if necessary; (2) Monthly supervision of townships by Laibin City during screening, with frequency adjusted as needed; (3) After each supervision check, the supervision team will write a report and provide feedback to the provincial project team.

3. Data Verification: On the day of screening completion, data managers will check for missing items or logical errors in the personal information forms completed on-site. Any issues will be corrected promptly the next day by contacting the screening officers.
